# Immunogenicity and safety of heterologous versus homologous prime-boost schedules with an adenoviral vectored and mRNA COVID-19 vaccine: a systematic review

**DOI:** 10.1186/s40249-022-00977-x

**Published:** 2022-05-13

**Authors:** Jingjing Lv, Hui Wu, Junjie Xu, Jiaye Liu

**Affiliations:** 1 Expanded Program Immunization Division of Shandong Provincial Center for Disease Control and Prevention, Shandong Provincial Key Laboratory of Infectious Disease Control and Prevention, Jinan, 250014 China; 2 Nosocomial Infection Control Department, Shenzhen University General Hospital, Shenzhen, 518071 China; 3 Clinical Research Academy, Peking University Shenzhen Hospital, Peking University, Shenzhen, 518036 China; 4 School of Public Health, Shenzhen University Health Science Center, No. 1066 Xueyuan Avenue, Shenzhen, 518060 China

**Keywords:** Homologous vaccination, Heterologous vaccination, COVID-19, Immunogenicity, Safety

## Abstract

**Background:**

Heterologous prime-boost with ChAdOx1 nCoV-19 vector vaccine (ChAd) and a messenger RNA vaccine (BNT or mRNA-1273) has been widely facilitating mass coronavirus disease 2019 (COVID-19) immunisation. This review aimed to synthesize immunogenicity and reactogenicity of heterologous immunisations with ChAd and BNT (mRNA-1273) vaccine compared with homologous ChAd or BNT (mRNA-1273) immunisation.

**Methods:**

PubMed, Web of Science, and Embase databases were searched from inception to March 7, 2022. Immunogenicity involving serum antibodies against different SAS-CoV-2 fragments, neutralizing antibody, or spike-specific T cells response were compared. Any, local and systemic reactions were pooled by meta-analysis for comparison.

**Results:**

Of 14,571 records identified, 13 studies (3024 participants) were included for analysis. Compared with homologous BNT/BNT vaccination, heterologous ChAd/BNT schedule probably induced noninferior anti-spike protein while higher neutralizing antibody and better T cells response. Heterologous ChAd/BNT (mRNA-1273) immunisation induced superior anti-spike protein and higher neutralizing antibody and better T cells response compared with homologous ChAd/ChAd vaccination. Heterologous ChAd/BNT (mRNA-1273) had similar risk of any reaction (*RR* = 1.30, 95% *CI*: 0.86−1.96) while higher risk of local reactions (*RR* = 1.65, 95% *CI*: 1.27−2.15) and systemic reactions (*RR* = 1.49, 95% *CI*: 1.17−1.90) compared with homologous ChAd/ChAd vaccination. There was a higher risk of local reactions (*RR* = 1.16, 95% *CI*: 1.03−1.31) in heterologous ChAd/BNT (mRNA-1273) vaccination compare with homologous BNT/BNT but a similar risk of any reaction (*RR* = 1.03, 95% *CI*: 0.79−1.34) and systemic reactions (*RR* = 0.89, 95% *CI*: 0.60−1.30).

**Conclusions:**

Heterologous ChAd/BNT schedule induced at least comparable immunogenicity compared with homologous BNT/BNT and better immunogenicity compared with homologous ChAd/ChAd vaccination. The synthetical evidence supported the general application of heterologous prime-boost vaccination using ChAd and BNT COVID-19 vaccines.

**Graphical Abstract:**

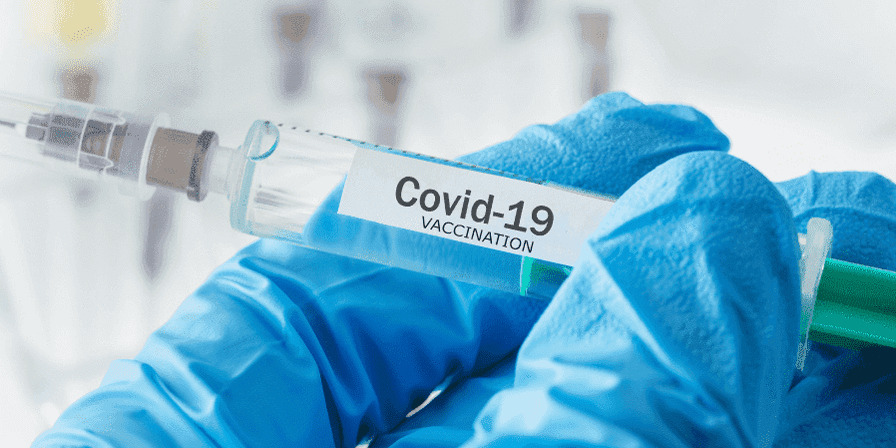

**Supplementary Information:**

The online version contains supplementary material available at 10.1186/s40249-022-00977-x.

## Background

As of March 12, 2022, severe acute respiratory syndrome virus 2 (SARS-CoV-2) infection has resulted in more than 452 million infections worldwide, with a total death toll of more than 6.0 million [1]. The patients with cancer had higher rates of coronavirus disease 2019 (COVID-19)-induced complications and mortality than the general population [2–4]. Frequently emerging vital mutations have raised significant concerns globally. Active immunization is the most efficient and vital strategy for fighting against this emerging infectious disease. Several vaccines with proven effectiveness are being deployed globally, including Pfizer-BioNTech’s mRNA COVID-19 vaccine (BNT) [5], Oxford–AstraZeneca’s adenovirus vectored vaccine (ChAd) [6], China’s Sinovac [7], and Sinopharm vaccines [8]. Mass vaccination raised hope for expeditious ending the COVID-19 pandemic.

BNT and ChAd have been the most widely used authorized COVID-19 vaccines worldwide. Due to a potentially higher risk of thromboembolic events in younger individuals [9], several European countries restricted their recommendations for ChAd COVID-19 vaccination to older individuals (e.g., older than 55 years in France and older than 60 years in Germany) [10, 11]. Heterologous boost immunisation with an mRNA vaccine was consequently recommended for younger individuals who had already received the first immunization with the ChAd COVID-19 vaccine. The changed recommendations contributed to several real-world studies to compare the immunogenicity and reactogenicity of heterologous ChAd/mRNA immunisation with homologous platform vaccines [12, 13]. Additionally, a randomised, controlled trial provided robust evidence on the safety and immunogenicity of heterologous versus homologous prime-boost schedules [14]. Although a majority of previous studies concluded that heterologous schedules incorporating vector vaccines and mRNA vaccines could induce comparable or superior humoral and cellular responses compared with homologous immunisation, the world health organization has not recommended heterologous prime-boost COVID-19 vaccination as an alternative strategy in the necessary settings. So far, there has been scarce pooled evidence on the safety and immunogenicity of heterologous COVID-19 vaccination, which is urgently needed for updating vaccination guidance worldwide.

Except for sufficing changes in guidance for vaccine usage, heterologous COVID-19 vaccination could also address shortages of vaccines to avoid delayed administration of the second dose and provide an alternative strategy for individuals who develop a contraindication to a specific vaccine after their first dose. Moreover, the emerging SARS-CoV-2 variants of concern have drawn attention, and breakthrough infections have been reported. Heterologous vaccination might induce an enhanced or more durable humoral or cellular immune response to combat COVID-19 variants [15]. In addition, it has been proven that the patients with cancer would have reduced or short-term COVID-19 vaccine efficacy [16, 17]. Heterologous vaccination might be an alternative strategy for improving immunogenicity in this particular population.

Therefore, we conducted this systematic review to pool the evidence on the immunogenicity and safety of heterologous versus homologous prime-boost vaccination schedule.

## Methods

### Literature search strategy

Relevant studies were searched in PubMed, Embase, and Web of Science from their inception to March 7, 2022 using a combination of comprehensive keywords, such as ‘COVID-19,’ ‘coronavirus disease 2019,’ ‘severe acute respiratory syndrome coronavirus 2,’ ‘SARS-CoV-2,’ ‘vaccination,’ and ‘vaccine’ with Boolean operators and MeSH terms. No constraints were placed on language. Besides, we searched relevant systematic reviews for additional papers. The process of searching, reviewing, selecting literature was independently performed by two authors. Discrepancies were resolved through consultation with a third author.

### Study selection criteria

Published papers were eligible for inclusion if they met the inclusion criteria: (1) studies were observational studies (prospective or retrospective cohort) or randomized trials with a minimum of ten adult participants in any subject group, (2) studies at least involved one type of heterologous prime-boost COVID-19 vaccination (i.e., mRNA vaccine boosting after ChAd vaccine priming, ChAd vaccine boosting after mRNA vaccine priming), (3) studies at least had one type of homologous prime-boost COVID-19 vaccination as the control group (i.e., mRNA vaccine boosting after mRNA vaccine priming, ChAd vaccine boosting after ChAd vaccine priming), (4) studies reported at least one of the outcomes of interest after boosting vaccination: serum antibodies against different SAS-CoV-2 fragments, and neutralizing antibody.

The exclusion criteria were as follows: (1) studies involved subjects who were ever or currently infected with SARS-CoV-2, (2) studies involved subjects with impaired immunity or immunosuppression, (3) studies involved subjects with severe diseases, such as patients who needed haemodialysis, (4) studies without baseline data reported, (5) studies were reviews.

### Data extraction

We extracted the data according to a standardized form: study characteristics (first author, year of publication, country of origin, and study design), subject characteristics (sample size, age, gender), vaccination strategies (priming vaccine, interval time between priming and boosting vaccination, boosting vaccine), and outcomes (interval time between boosting vaccination and outcomes evaluation, levels of serum antibodies against different SAS-CoV-2 fragments and neutralizing antibody, frequencies and phenotype of specific T cells and B cells, cytokine levels, and the number of adverse events). This process was also conducted by two authors independently and checked by a third author.

### Risk of bias assessment

We used the Newcastle–Ottawa Scale quality assessment scale for the quality assessment of cohort studies [18], which is comprised of three domains: selection, comparability, and outcome. The total score of the three domains is nine, with a score of 7−9 high quality (low risk of bias), 4−6 fair quality (moderate risk of bias), and 1−3 low quality (high risk of bias), respectively. In addition to cohort studies, we also included randomized controlled trials (RCTs) in this systematic review. The risk of bias in RCTs was assessed using version 2 of the Cochrane risk-of-bias tool for randomized trials [19].

### Outcome definitions

Primary outcomes were immunogenicity of heterologous and homologous COVID-19 vaccination, including anti-S1 IgG, anti-RBD IgG, anti-full spike IgG, ACE2–RBD binding inhibition (%), reciprocal titres of neutralizing antibodies, neutralization capacities, and neutralization inhibition (%) after boosting vaccination. The second outcomes included frequency and phenotype of spike-specific B cells and T cells, IFN γ-secreting T cells specific to spike protein epitopes, and local and systemic reactions after boosting vaccination.

### Statistical analysis

We tabulated the extracted information using Microsoft Excel version 2016 (Microsoft Office, CA, USA) spreadsheets and performed a meta-analytical evaluation using R 4.0.3. The techniques used to measure SARS-CoV-2 specific antibodies and criteria for positivity varied in different studies. Thus, meta-analysis was inappropriate to compare the immunogenicity of different studies. Instead, a qualitative description was mainly used to compare and pool the immunogenicity. Concerning adverse events (AEs) analysis, risk ratio (*RR*) and 95% confidence intervals (*CI*s) were used as the comparative index under evaluation. RR is estimated as the event rate in the trial group divided by the same rate in the control group. A random-effects, Mantel–Hanzeal model (95% *CI*) was used to determine effect sizes between studies. Statistical heterogeneity was assessed using the Cochrane *Q* test and *I*^2^ statistics. The assessment of potential publication bias by funnel plots and Egger’s test were expected; however, it was not done. The reason is that there were fewer than ten studies included in forest plot analysis. In this case, it is not recommended the funnel plots and Egger’s test because it could yield misleading results [20].

## Results

### Search results and characteristics of included studies

**Fig. 1:**
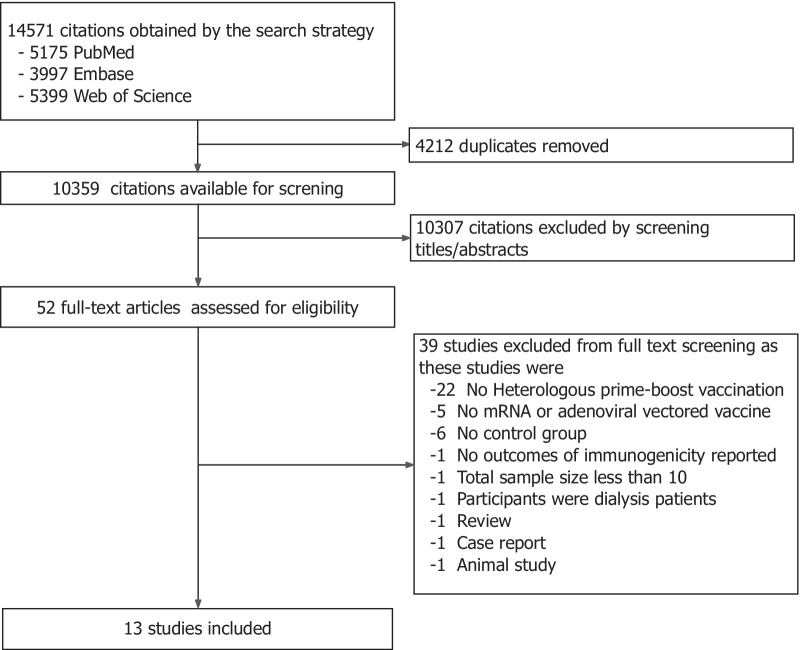
Study selection

As of March 7, 2022, the literature search initially identified 14,571 articles. We then selected articles according to the defined inclusion and exclusion criteria. Figure 1 depicts the process of study selection and reasons for exclusions. A total of 4212 duplicate articles in different databases were removed. We screened the title and abstract of the remaining articles and excluded ineligible articles. Finally, 52 full-text articles were further assessed for eligibility. Twenty-two studies were not included in our review because they don't use the heterologous prime-boost vaccination. Five studies without mRNA or adenoviral vectored COVID-19 vaccine, six studies without homologous prime-boost COVID-19 vaccination as control groups, one study without immunogenicity, one with a sample size less than ten, one with a pre-existing clinical disease, one review, one case report, and one animal study were excluded, respectively.

Finally, 13 studies [12–15, 21–29] from 52 articles were identified that fulfilled our inclusion and exclusion criteria, Nine studies were conducted in Germany [12, 13, 15, 21–24, 26, 27] two in UK [14, 29], and two in France [25, 28]. Of these studies, 12 were observational studies [12, 13, 15, 21–29] and one was randomized trial [14]. All studies involved ChAd/BNT (mRNA-1273) schedule and two study involved BNT/ChAd schedule [14, 24]. The characteristics of the included studies are summarized in Table 1.

**Table 1:** Main characteristics of included studies and subjects

First author	Year	Country	Design	Vaccination schedule	Prime-boost interval (days)	*N*	Age (years)	Male (n,%)	Boost-outcomes interval	Outcomes
Louise Benning [21]	2021	Germany	Prospective cohort	ChAd/ChAd	82 (82–83)^a^	17	55 (33–60)^a^	6 (35)	20 (19–21)^a^	Anti-S1 IgG, SARS-CoV-2 Neutralizing Antibodies, IgG antibodies against different SARS-CoV-2 target antigens
				ChAd/BNT	83 (77–84)^a^	35	30 (24–45)^a^	12 (34)	20 (19–21)^a^	
				BNT/BNT	20 (20–20)^a^	82	45 (33–56)^a^	19 (23)	20 (19–21)^a^	
Joana Barros-Martins [12]	2021	Germany	Retrospective cohort	ChAd/ChAd	73 (45–85)^a^	32	41 (21–64)^a^	12 (38)	16 (13–22)^a^	Anti-S IgG, Anti-S IgA, Neutralizing Antibodies, frequency and phenotype of B cells and T cells
				ChAd/BNT	74 (62–84)^a^	55	39 (22–61)^a^	15 (27)	17 (13–23)^a^	
				BNT/BNT	22 (18–28)^a^	21	38 (23–59)^a^	9 (43)	30 (15–65)^a^	
Xinxue Liu [14]	2021	UK	RCT	ChAd/ChAd	28	90^c^ 25^d^	57.6 (50.1–69.1)^ac^ 55.3 (50.7–64.1)^ad^	52 (58)^c^ 12 (48)^d^	28	Anti spike IgG, Normalised NT50(Live virus neutralising antibody), NT50(Pseudotype virus neutralising antibody), SFC per million PBMCs, Adverse events
				ChAd/BNT	28	90^c^ 24^d^	57.6 (50.1–69.1)^ac^ 58.9 (51.8–68.3)^ad^	50 (56)^c^ 15 (63)^d^	28	
				BNT/BNT	28	93^c^ 26^d^	57.7 (50.2–69.3)^ac^ 54.7 (50.1–67.2)^ad^	44 (47)^c^ 14 (54)^d^	28	
				BNT/ChAd	28	90^c^ 25^d^	56.1 (50.5–68.9)^ac^ 55.8 (51.4–67.0)^ad^	49 (54)^c^ 15 (60)^d^	28	
Alexandre Vallée [28]	2021	France	Retrospective, cross-sectional	ChAd/BNT	84 (3)^a^	130	37 (13)^a^	26 (20)	38 (7)^a^	Anti spike IgG
				BNT/BNT	27 (6)^a^	67	32 (11)^a^	8 (12)	42 (9)^a^	
Tina Schmidt [26]	2021	Germany	Cohort	ChAd/ChAd	10.8 ± 1.4week^b^	55	48.6 ± 11.9^b^	20 (36)	14 (2)^a^	Spike-specific IgG, neutralizing antibody, spike-specific CD4 and CD9 T cells, B cells, Adverse events
				ChAd/BNT	11.2 ± 1.3week^b^	97	40.8 ± 11.1^b^	26 (27)	14 (1)^a^	
				BNT/BNT	4.3 ± 1.1weeks^b^	64	44.7 ± 14.3^b^	18 (28)	14 (1.25)^a^	
David Hillus [13]	2021	Germany	Prospective cohort	ChAd/ChAd	83 (71–84)^a^	36^c^ 36^d^	51 (33–59)^ac^ 51 (33–59)^ad^	13 (36)^c^ 13 (36)^d^	24 (20–28)^a^	full spike-IgG, S1-IgG, RBD-IgG, neutralising antibody, IFN-γ
				ChAd/BNT	71 (70–73)^a^	104^c^ 94^d^	37 (29–51)^c^ 37 (29–48)^ad^	26 (25)^c^ 23 (24)^d^	21 (20–21)^a^	
				BNT/BNT	21 (21–21)^a^	159^c^ 101^d^	34 (29–43)^c^ 35 (30–47)^ad^	72 (45)^c^ 28 (28)^d^	28 (27–31)^a^	
Dorit Fabricius [15]	2021	Germany	Cohort	BNT/BNT	German guidelines	15	47 (26–64)^a^	3 (20)	14 or 21^a^	Neutralization capacities, anti-spike IgG titers, IFN-γ
				mRNA-1273/mRNA-1273	German guidelines	13	51 (34–61)^a^	5 (38)	14 or 21^a^	
				ChAd/BNT	German guidelines	26	44 (22–64)^a^	24 (92)	14 or 21^a^	
				ChAd/mRNA-1273	German guidelines	10	33 (21–47)^a^	3 (30)	14 or 21^a^	
Matthias Tenbusch [27]	2021	Germany	Cohort	ChAd/BNT	63 (63–77)^a^ 63 (63–63)^a^	232^e^ 250^e^	47 (33–55)^ae^ 52 (31–59)^a^	42 (18)^e^ 90 (36)^e^	14 (13–15)^ad^ 14 (14)^ad^	Surrogate neutralization activity
				BNT/BNT	21 (21–22)^a^ 23 (21–25)^a^	410^e^ 127^e^	38 (31–48)^ae^ 41 (27–52)^ae^	162 (40)^e^ 37 (29)^e^	14 (13–15)^ad^ 15 (13–15)^ad^	
				ChAd/ChAd	63 (63–63)^a^	66^e^	57 (45–62)^ae^	23 (35) ^e^	15 (13–15)^a^	
Swantje I. Hammerschmidt [23]	2021	Germany	Cohort	ChAd/ChAd	68 (45–91)^a^	31	NR	11 (35)	17 (13–23)^a^	Reciprocal titers of Neutralizing Antibodies
				ChAd/BNT	68 (45–91)^a^	54	NR	14 (26)	17 (13–23)^a^	
				BNT/BNT	21 (18–27)^a^	30	NR	9 (30)	30 (15–65)^a^	
Rudiger Gross [22]	2022	Germany	Retrospective cohort	ChAd/BNT	56^a^	26	31 (25–46)^a^	10 (38)	14–19^a^	Anti-spike-IgM and IgG, surrogate virus neutralization, 50% pseudovirus neutralization, IFN-γ, IFN-α, IL-2
				BNT/BNT	21^a^	14	42 (25–65)^a^	10 (71)	13–15^a^	
Bruno Pozzetto [25]	2021	France	Cohort	ChAd/BNT	85 (84–85)^a^	29	34 (27–40)^a^	9 (31)	30 (28–34)^a^	RBD and spike S1-specific IgG, IgA, neutralizing activity
				BNT/BNT	29 (26–31)^a^	31	41 (33–52)^a^	8 (26)	28 (27–31)^a^	
Samantha J Westrop [29]	2022	UK	Contemporaneous cohort	ChAd/BNT	73 (64–83)^a^	237	47 (37–59)^a^	55 (23)	30 (21–39)^a^	IgG antibody levels against the SARS-CoV-2 spike protein (S-antibody) and Nucleoprotein (N-antibody)
				BNT/BNT	76 (70–76)^a^	135	71 (69–72)^a^	64 (47)	30 (21–39)^a^	
				ChAd/ChAd	70 (54–77)^a^	121	65 (54–69)^a^	59 (49)	30 (21–39)^a^	
				BNT/ChAd	79 (65–99)^a^	123	51 (40–63)^a^	36 (29)	30 (21–39)^a^	
Swantje I. Hammerschmidt [24]	2022	Germany	Cohort	ChAd/mRNA-1273	80 (62–87)^a^	42	38 (19–66)^a^	13 (31)	14	Neutralizing antibodies against the Delta variant
				mRNA-1273/mRNA-1273	29 (27–42)^a^	24	34 (23–63)^a^	11 (44)	14	
				ChAd/ChAd	78 (35–93)^a^	38	47 (24–69)^a^	15 (39)	14	

Data in columns of “N”, “Age”, and “Male” represented the number of whole cohorts, we add special annotation (i.e. c, d, e) if data represented number of immunology cohorts or one study included more than one cohort

*Ab* antibody, *BNT* BNT162b2 vaccine, Pfizer–BioNTech, *ChAd* ChAdOx1 nCoV-19 vaccine, AstraZeneca, *NR* not reported, *RBD* receptor-binding domain, *NT*_*50*_ 50% neutralising antibody titre, *SFC* spot-forming units, *PBMC* peripheral blood mononuclear cell, *IFN-γ* interferon-γ

^a^Data were presented as the median, median (Q1, Q3), or median (interquartile range)

^b^Data were presented as the mean ± standard deviation

^c^Data represented the number of whole cohorts

^d^Data presented the number of immunology cohorts

^e^Data presented the number of each cohort included in the study

The quality assessment scores for included cohorts are shown in Additional file 1: Table S1. Overall, four high-quality cohort studies [22, 25, 28, 29] and eight fair-quality cohort studies [12, 13, 15, 21, 23, 26, 27, 30] were included in this systematic review. The randomized trial [14] was considered to have a low risk of bias.

### Characteristics of various prime-boost vaccination strategies

In this systematic review, prime-boost vaccination schedules include ChAd/BNT (mRNA-1273) and BNT/ChAd. All studies involved ChAd/BNT (mRNA-1273) heterologous schedule and had BNT/BNT (mRNA-1273) as the control group, and meanwhile, ten of which also had ChAd/ChAd as the control group [12–15, 21, 23, 24, 26, 27, 29].

### Effect of immunogenicity with heterologous strategy on outcomes

#### ChAd/BNT(mRNA-1273) vs BNT/BNT

Thirteen studies [12–15, 21–29] involved 14 times of comparisons between ChAd/BNT(mRNA-1273) heterologous schedule and homologous BNT/BNT (mRNA-1273/mRNA-1273), four of them studies reported comparative efficacy on anti-RBD IgG and all of them did not find significant difference on this outcome [13, 21, 25, 26]. Eight studies [12–14, 21, 22, 25, 28, 29] reported comparative effectiveness on anti-S protein IgG, seven of them [12–14, 21, 25, 28, 29] showed comparable efficacy on this indicator, and one of them [22] found that ChAd/BNT induced higher cumulative anti-spike-IgM and IgG concentrations. Ten studies [13–15, 21–27] supplied 11 times of comparison on neutralizing antibody, six of them [13, 15, 22, 25–27] with seven times of comparison found that ChAd/BNT(mRNA-1273) heterologous schedule could induce better response on neutralizing antibody involving against B.1.351 variant and B.1.1.7 variant, two of them [14, 21] found similar response, and two of them [23, 24] found a lower reciprocal titres of neutralizing antibody against Delta variant. Four studies [13, 15, 25, 26] explored spike-specific IFN-γ secretion, three of them [13, 15, 26] discovered that heterologous ChAd/BNT(mRNA-1273) immunisation could induce higher spike-specific IFN-γ secretion, and one of them found similar whole-blood IFN-γ [25] (Table 2). In conclusion, it seemed that the majority of studies indicated heterologous ChAd/BNT(mRNA-1273) immunisation schedule induced superior or at least comparable humoral and cellular response against SARS-CoV-2 compared with homologous BNT/BNT immunisation schedule, while definite conclusion has yet been reached on the response against variants.

**Table 2:** Comparison of different prime-boost immunization strategies on immunogenicity in included studies

Comparison^a^	First author	RBD Ab^b^	Spike protein Ab^b^	Neutralizing Ab^b^	T cell response and others^b^	Conclusion
ChAd/BNT vs BNT/BNT	Louise Benning [21]	NS in MFI values	Higher MFI of full spike protein (24,243 vs 23,849), S1 protein (19,332 vs 16,955), and S2 protein (13,138 vs 9696) values Comparable anti-S1 IgG levels (116.2 to 145.5) dimensionless index	NS in inhibition of RBD-ACE2 binding (96.8% vs 97.0%)	NR	ChAd/BNT superior to BNT/BNT in spike protein Ab, while comparable in RBD and neutralizing Ab
ChAd/BNT vs BNT/BNT	Joana Barros-Martins [12]	NR	NS in Anti-S IgG (625.7 vs 303.2 RU/ml by quantitative ELISA) and IgA (3.76 vs 2.56 ratio)	NR	NR	ChAd/BNT comparable to BNT/BNT in Anti-S IgG and IgA
ChAd/BNT vs BNT/BNT	Xinxue Liu [14]	NR	NS in Anti-S IgG levels (12,995 vs 13,938 ELU/mL by ELISA)	NS in PNA NT50 (515 vs 574)	NR	ChAd/BNT comparable to BNT/BNT in Anti-S IgG levels and PNA NT50
ChAd/BNT vs BNT/BNT	Alexandre Vallée [28]	NR	NS in S protein IgG levels (7268.6 vs 10,734.9 RLU by CMIA)	NR	NR	ChAd/BNT comparable to BNT/BNT in S protein IgG
ChAd/BNT vs BNT/BNT	David Hillus [13]	NS in anti-RBD IgG (5.6 vs 5.4S/Co by solid phase immunoassay)	NS in anti-full S and anti-S1 IgG Higher Anti-S1 IgG avidity index (93.6% vs 73.9%)	NS in ACE2–RBD binding inhibition (97.1% to 96.6%) Higher serum neutralising activity (ID50 against to alpha variant 956.6 vs 369.2, ID50 against to beta variant 417.1 vs 72.4)	Higher S-specific T-cell responses (IFN-γ: 4762 vs 2026 mIU/mL)	ChAd/BNT superior to BNT/BNT in serum neutralising activity and S-specific T-cell responses; Comparable in anti-RBD IgG, anti-full S, anti-S1 IgG and anti-S1 IgG avidity
ChAd/BNT vs BNT/BNT	Dorit Fabricius [15]	NR	NR	Higher neutralization capacities against wildtype RBD and B.1.1.7 variant (82% to 63%)	Higher IFN-γ secretion	ChAd/BNT superior to BNT/BNT in neutralization capacities and T cells responses
ChAd/BNT vs BNT/BNT	Matthias Tenbusch [27]	NR	NR	Higher surrogate neutralisation activity (IC50: 3377 to 1789AU/mL)	NR	ChAd/BNT superior to BNT/BNT in neutralisation Ab
ChAd/BNT vs BNT/BNT	Swantje I. Hammerschmidt [23]	NR	NR	Lower reciprocal titers of neutralizing against Delta (180 to 540)	NR	ChAd/BNT inferior BNT/BNT in neutralisation Ab
ChAd/(BNT or mRNA-1273) vs (BNT or mRNA-1273)/(BNT or mRNA-1273)	Tina Schmidt [26]	NS in IgG to RBD of S protein (3630 vs 4932 BAU/mL by ELISA)	NR	Higher in inhibition of ACE2-S1 RBD (100.07% to 99.68%)	Higher percentages of spike-specific IFN-γ-producing CD8 T cells levels (0.28% to 0.06%) NS in CD4 T cells levels (0.17% to 0.16%)	ChAd/BNT superior to mRNA-1273 in neutralizing Ab and CD8 T cells levels, while comparable in RBD Ab and CD4 T cells levels
ChAd/mRNA-1273 vs BNT/BNT	Dorit Fabricius [15]	NR	NR	Higher neutralization capacities against wildtype RBD and B.1.351 variant (85% to 59%), B.1.1.7 variant (87% to 63%)	NR	ChAd/mRNA-1273 superior to BNT/BNT in neutralization capacities
Comparison	First Author	RBD Ab	Spike protein Ab	Neutralizing Ab	T cell response and others	Conclusion
ChAd/BNT vs BNT/BNT	Rudiger Gross [22]	NR	8.1-fold higher quantified cumulative anti-spike-IgM and IgG concentrations (8815 vs 1086 U/ml)	3.9-fold higher neutralizing activity correlated with IgG or IgM/G titres (2744 vs 709)	Levels of spike-specific CD8 + T cells producing IL-2 in agreement with BNT/BNT	ChAd/BNT superior to BNT/BNT in spike IgM and IgG and neutralizing activity, while comparable in T cell response
ChAd/BNT vs BNT/BNT	Bruno Pozzetto [25]	NS in positivity rate of RBD IgG (both 100%)	NS in positivity rate of spike S1-specific IgG (both 100%); NS in S1-specific IgA levels (37.4 vs 46.7 ng/ml)	Higher neutralizing efficacy (99% vs 62%); 2.3-fold to 3.6-fold higher serum neutralizing antibody titres against different variants	Two fold higher in frequency of RBD-binding mBCs; higher in IgD^–^CD27^+^ (67% vs 47%); lower in IgG-switched mBCs (48% vs 62%); three times higher in proportions of CD21^–^CD11c^+^ subset; higher in proportions of frequencies of activated RBD-specific mBCs; similar in whole-blood IFNγ (0.43 vs 0.33 UI/ml)	ChAd/BNT superior to BNT/BNT in neutralizing efficacy, T cell response, and B cell activation
ChAd/BNT vs BNT/BNT	Samantha J Westrop [29]	NR	NS in anti-S antibody level (6233 vs 5377), adjusted GMR:1.11	NR	NR	ChAd/BNT inferior to BNT/BNT in anti-S IgG levels
ChAd/mRNA-1273 vs mRNA-1273/mRNA-1273	Swantje I. Hammerschmidt [24]	NR	NR	Lower in neutralizing antibodies against the Delta variant (540 vs 1620)	NR	ChAd/mRNA-1273 inferior to mRNA-1273/mRNA-1273 in neutralizing antibodies against Delta variant
ChAd/BNT vs ChAd/ChAd	Louise Benning [21]	NR	Higher anti-S1 IgG levels (116.2 vs 13.1) by dimensionless index (CLIA)	Higher (96.8% vs 93.5%) in inhibition of RBD-ACE2 binding	NR	ChAd/BNT superior to ChAd/ChAd in S1 protein and neutralizing Ab
ChAd/BNT vs ChAd/ChAd	Joana Barros-Martins [12]	NR	Higher Anti-S IgG (625.7 vs 160.9 RU/ml by quantitative ELISA) and IgA (3.76 vs 0.87 ratio)	Higher reciprocal titers of neutralizing antibodies against Wuhan (4840 vs 540), B.1.1.7 (540 vs 20), P.1 (60 vs 0) and B.1.351 (60 vs 0) variants	Higher in spike-specific CD4 + and CD8 + T cells Higher in IFN-γ concentration NS in spike-specific memory B cells NS in TNF-α concentration	ChAd/BNT superior to ChAd/ChAd in S protein, neutralizing Ab and T cellular response, while comparable in memory B cells and TNF-α
ChAd/BNT vs ChAd/ChAd	Xinxue Liu [14]	NR	Higher in Anti-S IgG levels (12,995 vs 1387 ELU/mL by ELISA), GMR: 9.3	Higher in MNA NT50 titer (1269 vs 210), PNA NT50 titer (515 vs 61), GMR: 6.4 for MNA NT50 and 8.5 for PNA NT50	Higher in T-cell ELISpot, SFC per million PBMCs (184 vs 48), GMR: 3.9	ChAd/BNT superior to ChAd/ChAd in anti-S IgG levels, MNA NT50, PNA NT50, and cellular responses
ChAd/BNT vs ChAd/ChAd	David Hillus [13]	Similar in anti-RBD IgG (5.6 vs 4.9 S/Co by solid phase immunoassay)	Higher Anti-S1 IgG avidity index (93.6% vs 71.7%) NS in anti-full S and anti-S1 IgG	Higher ACE2–RBD binding inhibition (97.1% vs 92.4%) Higher serum neutralising activity (ID50 against to alpha variant 956.6 vs 212.5, ID50 against to beta variant 417.1 vs 48.5)	Higher S-specific T-cell responses (IFN-γ:4762 vs 1061 mIU/mL)	ChAd/BNT superior to ChAd/ChAd in anti-S1 IgG avidity, serum neutralising activity and S-specific T-cell responses, while comparable in anti-RBD IgG, anti-full S and anti-S1 IgG
Comparison	First Author	RBD Ab	Spike protein Ab	Neutralizing Ab	T cell response and others	Conclusion
ChAd/BNT vs ChAd/ChAd	Dorit Fabricius [15]	NR	Higher Anti-S1 IgG and IgA	Higher neutralization capacities against wildtype RBD and B.1.1.7 variant (82% vs 48%), B.1.351 variant (70% vs 57%), P.1 variant (55% vs 15%)	Higher IFN-γ secretion	ChAd/BNT superior to ChAd/ChAd T in neutralization capacities and T cells responses
ChAd/BNT vs ChAd/ChAd	Matthias Tenbusch [27]	NR	NR	Higher surrogate neutralisation activity (IC50: 3377 vs 106 AU/mL)	NR	ChAd/BNT superior to ChAd/ChAd in neutralisation Ab
ChAd/BNT vs ChAd/ChAd	Swantje I. Hammerschmidt [23]	NR	NR	Higher reciprocal titers of neutralizing against Delta (180 vs 20)	NR	ChAd/BNT superior to ChAd/ChAd in neutralisation Ab
ChAd/BNT or mRNA-1273 vs ChAd/ChAd	Tina Schmidt [26]	Higher IgG levels to RBD of S protein (3630 vs 404 BAU/mL by ELISA)	NR	Higher in inhibition of ACE2-S1 RBD (100.07% vs 83.37%)	Higher percentages of spike-specific IFN-γ-producing CD8 T cells levels (0.28% vs 0.04%) and CD4 T cells levels (0.17% vs 0.04%)	ChAd/BNT or mRNA-1273 superior to ChAd/ChAd in RBD Ab, neutralizing Ab, CD4 T cells, and CD8 T cells levels
ChAd/BNT vs ChAd/ChAd	Samantha J Westrop [29]	NR	Higher in anti-S antibody level (6233 vs862), adjusted GMR:6.29	NR	NR	ChAd/BNT superior to ChAd/ChAd in anti-S antibody
ChAd/mRNA-1273 vs ChAd/ChAd	Swantje I. Hammerschmidt [24]	NR	NR	Higher in neutralizing antibodies against the Delta variant(540 vs 20)	NR	ChAd/mRNA-1273 superior to ChAd/ChAd in neutralizing antibodies against Delta variant
BNT/ChAd vs BNT/BNT	Xinxue Liu [14]	NR	Lower anti-S IgG levels (7133 vs 13,938 ELU/mL by ELISA), GMR:0.51	Lower PNA NT50 titer (383 vs 574), GMR:0.67	NS in SFC per million PBMCs, T-cell ELISpot (90 vs 81), GMR: 1.2	BNT/ChAd inferior to BNT/BNT in anti-S IgG levels and PNA NT50, while comparable in T cellular responses
BNT/ChAd vs BNT/BNT	Samantha J Westrop [29]	NR	NS in anti-S antibody level (4776 vs 5377), adjusted GMR:0.80	NR	NR	BNT/ChAd inferior to BNT/BNT in anti-S IgG levels
BNT/ChAd vs ChAd/ChAd	Xinxue Liu [14]	NR	Higher anti-S IgG levels (7133 vs 1387ELU/mL by ELISA)	Higher PNA NT50 titer (383 vs 61)	NR	BNT/ChAd superior to ChAd/ChAd in anti-S IgG levels and PNA NT50
BNT/ChAd vs ChAd/ChAd	Samantha J Westrop [29]	NR	Higher in anti-S antibody level (4776 vs862), adjusted GMR:4.55	NR	NR	BNT/ChAd superior to ChAd/ChAd in anti-S antibody

*Ab* antibody, *BNT* BNT162b2 vaccine, Pfizer–BioNTech, *ChAd* ChAdOx1 nCoV-19 vaccine, AstraZeneca, *NR* not reported, *MFI* mean fluorescence intensity, *RBD* receptor-binding domain, *ACE2* angiotensin-converting enzyme 2, *PNA* pseudotype virus neutralization assay, *NT*_*50*_ 50% neutralising antibody titre, *CMIA* chemiluminescent microparticle immunoassay, *ID50* 50% inhibitory dilutions, *IFN-γ* interferon-γ, *IC50* inhibitory 50% concentration, *ELISA* enzyme-linked immunosorbent assay, *GMR* geometric mean ratio, *NT50* 50% neutralising antibody titre, *SFC* spot-forming units, *PBMC* peripheral blood mononuclear cell, *mBCs* memory B cells

^a^Studies in the table were arranged by the types of prime-boost immunization strategies. Some studies may include more than one type of prime-boost immunization strategy or more than one comparative group, so these studies were presented in more than one row

^b^All the comparisons in the table indicate the value of heterologous prime-boost vaccination vs that of homologous prime-boost vaccination

#### ChAd/BNT(mRNA-1273) vs ChAd/ChAd

Of the ten studies [12–15, 21, 23, 24, 26, 27, 29] involved ChAd/BNT (mRNA-1273) heterologous schedule with homologous ChAd/ChAd as a control group, two studies [13, 26] reported comparative efficacy on anti-RBD IgG, one of which showed similar and another showed higher RBD IgG in ChAd/BNT(mRNA-1273) groups compared with that in homologous ChAd/ChAd groups. Six studies [12, 14, 15, 21, 26, 29] reported comparative efficacy on anti-S protein IgG, all of which showed a higher anti-S IgG level in ChAd/BNT (mRNA-1273) groups compared with that in homologous ChAd/ChAd groups. Nine studies [12–15, 21, 23, 24, 26, 27] reported comparative efficacy on neutralizing antibody and found better responses in ChAd/BNT (mRNA-1273) groups compared with homologous ChAd/ChAd groups; especially, two study [15, 24] found that ChAd/BNT (mRNA-1273) heterologous schedule could induce better response on neutralizing antibody capacities against B.1.351 variant, B.1.1.7 variant, or P.1 variant compared with that in homologous ChAd/ChAd groups. Four studies [12, 13, 15, 26] explored spike-specific T-cell-mediated immune response and all of them [12, 13, 15, 26] indicated that heterologous ChAd/BNT(mRNA-1273) could induce higher spike-specific IFN-γ secretion compared with homologous ChAd/ChAd groups (Table 2). Overall, heterologous ChAd/BNT (mRNA-1273) immunisation schedule induced superior humoral and cellular response against SARS-CoV-2 compared with homologous ChAd/ChAd immunisation schedule.

#### BNT/ ChAd vs BNT/BNT or ChAd/ChAd

Two studies [14, 29] investigated the difference of specific immune response between heterologous BNT/ChAd and homologous BNT/BNT vaccination, one of which found that heterologous BNT/ChAd induced inferior anti-S IgG and neutralizing antibody while similar T cell response compared with homologous BNT/BNT vaccination, the other found similar response in anti-S rotein IgG. Meanwhile, the two studies also compared the immune response between heterologous BNT/ChAd and homologous ChAd/ChAd vaccination, both of which found heterologous BNT/ChAd schedule induced higher anti-S IgG and one study also found heterologous BNT/ChAd schedule induced higher neutralizing antibody compared with homologous ChAd/ChAd schedule (Table 2).

### Adverse events with heterologous strategy on outcomes

**Fig. 2:**
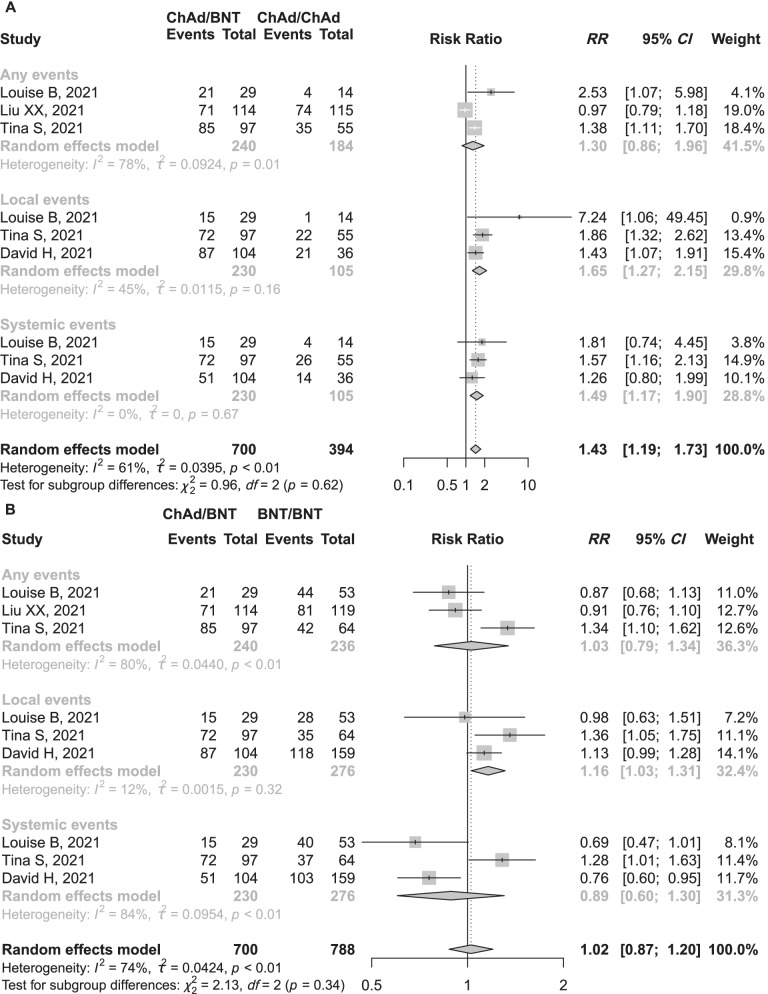
Estimates of risk ratio of reactions stratified by any, local, and systematic reactions. Figure shows the risk ratio of any, local, and systemic events in participants who received heterologous ChAd/BNT schedule compared with those in homologous ChAd/ChAd (**A**) and the risk ratio of any, local, and systemic events in participants who received heterologous ChAd/BNT schedule compared with those in homologous BNT/BNT (**B**). *BNT* BNT162b2 vaccine, Pfizer–BioNTech, *ChAd* ChAdOx1 nCoV-19 vaccine, AstraZeneca, *CI* confidence intervals, *RR* risk ratio

Four studies [13, 14, 21, 26] compared AEs incidences between heterologous ChAd/BNT (mRNA-1273) and homologous BNT/BNT or ChAd/ChAd vaccination. All of them used standardized questionnaires to collected AEs after vaccination (Additional file 1: Table S2). We conducted a meta-analysis to compare the frequencies of any reaction, local reaction and systemic reaction after prime-boost vaccination. There was a similar risk of any reaction (*RR* = 1.30, 95% *CI*: 0.86−1.96, *I*^2^ = 78%) while a higher risk of local reactions (*RR* = 1.65, 95% *CI*: 1.27−2.15, *I*^2^ = 45%) and systemic reactions (*RR* = 1.49, 95% *CI*: 1.17−1.90, *I*^2^ = 0%; Fig. 2A) in heterologous ChAd/BNT (mRNA-1273) groups compared with that in homologous ChAd/ChAd group. Similarly, there was a higher risk of local reactions (*RR* = 1.16, 95% *CI*: 1.03−1.31, *I*^2^ = 12%) in heterologous ChAd/BNT (mRNA-1273) vaccination compared with homologous BNT/BNT but similar risk of any reaction (*RR* = 1.03, 95% *CI*: 0.79−1.34, *I*^2^ = 80%) and systemic reactions (*RR* = 0.89, 95% *CI*: 0.60−1.30, *I*^2^ = 84%; Fig. 2B). One study reported the frequencies of severe adverse events (SAE) were 9.5% in ChAd/ChAd, 11.3% in ChAd/BNT, 1.2% in BNT/BNT, and 7.8% in BNT/ChAd, respectively [14]. Another study reported the frequencies of severe local adverse events were 4% in BNT/BNT, 3% in ChAd/ChAd, and 7% in ChAd/BNT, respectively; the frequencies of severe systemic adverse events were 6% in BNT/BNT, 6% in ChAd/ChAd, and 2% in ChAd/BNT, respectively [13]. However, statistic tests were not conducted in the frequencies of SAE between heterologous and homologous vaccination in the two studies. Based on these crude data, the frequencies of SAE seemed to be comparable between heterologous and homologous vaccination.

#### Heterogeneity

The *P* value for Cochrane’s Q test suggested high heterogeneity across studies for ChAd/BNT (mRNA-1273) vs BNT/BNT strategy in the assessment of any events and systematic and studies for ChAd/BNT (mRNA-1273) vs ChAd/ChAd strategy in any events assessment (*P* < 0.01, Fig. 2). Potential publication bias was not assessed because the number of studies was small (< 10) in all of the above meta-analyses.

## Discussion

Equitable access to safe and effective vaccines is critical to ending the COVID-19 pandemic. In the situation of vaccines shortage and observed higher risk of severe adverse events for some subgroups after vaccination, optimizing the vaccination based on available COVID-19 vaccines is urgently needed. Our systematic review showed robust immunogenicity of heterologous prime-boost immunisation with ChAd and BNT. Compared with homologous BNT/BNT vaccination, heterologous ChAd/BNT (mRNA-1273) schedule probably induced non-inferior anti-spike protein while higher neutralizing antibody and better T cells response. Heterologous ChAd/BNT (mRNA-1273) immunisation induced superior anti-spike protein and higher neutralizing antibody and better T cells response compared with homologous ChAd/ChAd vaccination. Reactogenicity was tolerable in heterologous ChAd/BNT compared with homologous ChAd/ChAd or BNT/BNT vaccination. In addition, heterologous BNT/ChAd vaccination schedule showed weaker immunogenicity than homologous BNT/BNT vaccination. The robust immunogenicity elicited by heterologous ChAd/BNT vaccination schedule provides evidence for the feasibility of this promising vaccination strategy.

Antibodies to S protein, virus neutralization tests (VNT), pseudovirus neutralization tests (pVNT), and competitive neutralization tests (cVNT) of SARS-CoV-2 have been the most common antibody testing for evaluating the immune response of COVID-19 vaccination. The detecting methods of SARS-CoV-2 anti-spike (RBD or S) IgG and neutralizing antibodies were various in the included articles (Additional file 1: Table S3), which made the quantitative analysis of immunogenicity by meta-analysis unavailable. A significant number of studies have established associations between humoral responses and vaccine efficacy, such as against symptomatic diseases, severe diseases, and hospitalisation [31, 32]. Based on the above indicators, this systematic review found that heterologous ChAd/BNT vaccination induced humoral responses at least as high as or even better than those induced after homologous ChAd/ChAd or BNT/BNT schedule. The mechanisms of immune response induced by heterologous prime-boost vaccination are incompletely understood. Several factors, including the selection of antigen, type of vector, adjuvant, the order of vector injection, and the intervals between different vaccinations, influence the responses of prime-boost immunization [33]. Although neutralization against P.1, B.1.1.7, and B.1.351 induced both by ChAd and BNT were reduced, the protective efficacy against symptomatic COVID-19 caused by variants differed between homologous ChAd/ChAd and BNT/BNT schedule. [34–36] Thus, heterologous ChAd/BNT vaccination was expected to induce more robust immune responses against novel viral variants. Indeed, two studies in this review confirmed heterologous ChAd/BNT vaccination induced higher titres of neutralizing antibodies against P.1, B.1.1.7, and B.1.351 variants [12, 15]. In the setting of vaccine shortage and rapid expanding variants, heterologous ChAd/BNT vaccination might be a promising vaccination schedule against COVID-19 pandemic, beyond passive substitution vaccination due to rare severe adverse events.

The quantity and function of T-cell responses play a crucial part in the prognostication of COVID-19 and monitoring immune responses to SARS-CoV-2 vaccination and population-based immunity to SARS-CoV-2 variants of interest [37]. One study evaluated the immune response of homologous and heterologous mRNA and vector-based COVID-19 vaccine schedules in solid organ transplant recipients. It showed that cellular immunity was more frequently found (64.7%) than humoral response (35%), which indicated that assessment of antibodies was insufficient to identify COVID-19-vaccine responders [38]. Among ten included studies in this systematic review, five studies [12–15, 26] compared the spike-specific CD4 or CD8 T cell response and all these studies found that heterologous ChAd/BNT vaccination induced better spike-specific T cell response. Early responses to vaccination are important for shaping both humoral and cellular protective immunity. Increased interferon-gamma (IFN-γ) levels early after boost correlated with spike antibody levels, implying IFN-γ as a valuable biomarker of effective humoral immunity development in response to vaccination [39]. Specially, four studies [12, 13, 15, 26] in this systematic review showed a higher IFN-γ secretion in heterologous ChAd/BNT vaccination groups. Together with robust humoral and cellular responses, this review concluded heterologous ChAd/BNT vaccination could induce a broader immune response.

Because of rare but evidenced severe adverse events after vaccination with ChAd COVID-19 vaccine, heterologous ChAd/BNT vaccination has become the most common heterologous schedule. Nevertheless, heterologous vaccination with the reverse sequential schedule or other platforms might be needed in real-world immunization practice. One study included in this review showed that heterologous BNT/ChAd vaccination induced inferior anti-S IgG and neutralizing antibody compared with homologous BNT/BNT schedule while superior responses compared with homologous ChAd/ChAd vaccination [14, 40]. This result implied that heterologous BNT/ChAd is not an optimal sequential vaccination schedule taking no account of supply shortages and contraindications to prime vaccine. Further studies are needed to explore the immune response of various heterologous prime-boost immunization.

Decreases over time of vaccine-induced neutralising antibodies against SARS-CoV-2 have been observed with several COVID-19 vaccines [40, 41]. Moreover, many countries are experiencing a resurgence of COVID-19 mainly due to variants of SARS-CoV-2. In response, considering the administration of the third dose of COVID-19 vaccine as a booster dose has been the research interest for addressing potential waning immunity over time and reduced effectiveness against the delta variant. One study found that heterologous two BBIBP/BNT could induce higher anti-S IgG titre compared with the homologous schedule in BNT/BNT vaccination [30]. Consistent with this study, a third dose of the BNT COVID-19 vaccine after homologous BNT/BNT in Israel [42], a third dose of CoronaVac after homologous CoronaVac/CoronaVac in China [43], and seven COVID-19 vaccines as a third dose (booster) following two doses of ChAd COVID-19 or BNT in the UK [44] all resulted in a remarkable increase in the concentration of antibodies or increase in effectiveness for preventing severe COVID-19 outcomes. These results implied that a heterologous third dose after homologous prime-boost vaccination could be an alternative immuniz ation schedule.

A longer prime-boost interval is reported to induce a higher post-boost SARS-CoV-2 anti-spike IgG both for ChAd/ChAd [43] and for BNT/BNT [45]. Of the ten studies [12–15, 21, 23, 24, 26, 27, 29] involved ChAd/BNT (mRNA-1273) heterologous schedule with homologous ChAd/ChAd as a control group, the prime-boost intervals were similar between the two schedules. Thus, the difference on the immunogenicity probably attributed to the vaccination schedules. However, of the 13 studies [12–15, 21–29] involved ChAd/BNT (mRNA-1273) heterologous schedule with homologous BNT/BNT (mRNA-1273/mRNA-1273) as a control group, only two studies [14, 29] had comparable prime-boost intervals between the two schedules. Both the two studies showed the comparable immunogenicity between heterologous and homologous vaccination. However, for majority of included studies involved comparison between ChAd/BNT (mRNA-1273) and BNT/BNT (mRNA-1273/mRNA-1273), both prime-boost interval and vaccination schedule became the main confounders in the analysis of immunogenicity after boost vaccination. In addition, age, sex, race, and individual immune status also affected the comparison of immunogenicity between the heterologous and homologous vaccination. Moreover, there has not been studies directly comparing immunogenicity between different prime-boost intervals in single heterologous arm. Thus, whether prime-boost interval affects immunogenicity awaits future studies.

One of the most important purposes of heterologous ChAd/BNT vaccination was to address the high risk of thromboembolic events for patients who would receive homologous ChAd/ChAd vaccination. As expected, none of the thromboembolic events and other vaccination related severe events were observed in heterologous ChAd/BNT vaccination groups from included studies. Our systematic review found that heterologous prime-boost vaccination leads to a slightly higher risk of local reactions and systemic reactions compared with homologous ChAd/ChAd vaccination and a higher risk of local reactions compared with homologous BNT/BNT, which mainly resulted from the study of Tina Schmidt, et al. conducted in Germany [26]. This study showed that the reactions after the second dose were mainly determined by the severity of the priming vector vaccines. Despite that, heterologous boosting was well tolerated and comparable to homologous mRNA boosting. In addition, study design, study population demographics, and collection methods of adverse events could lead to differences in the assessment of this outcome.

This systemati c review provided a higher level of evidence on the immunogenicity and safety in heterologous prime-boost schedules with an adenoviral vectored and mRNA COVID-19 vaccine. This promising schedule provide an alternative strategy not only for relieving the shortage of vaccines but also for combating various variants during the global pandemic. Nevertheless, several issues are needed to be addressed in future. Firstly, our systematic review only included two kinds of COVID-19 vaccine with different technical routes. More studies especially high qualitied randomized controlled trials on heterologous and homologous schedules with other technical routes, e.g. inactivated, protein subunit could be conducted for providing more flexibility for future vaccination strategies. Furthermore, direct comparisons of immunogenicity and safety between different heterologous prime-boost schedules are also necessary. Secondly, the influence of prime-boost interval, race, and other potential confounders on immunogenicity should be evaluated for identifying the independent role of heterologous vaccination. Thirdly, some particular population subgroups (e.g. immunocompromised individuals, cancer patients, haemodialysis patients, etc.) have weaker immunogenicity compared with that in general population. Thus, a strengthening strategy is essential for these particular population. Whether heterologous vaccination could elicit robust immune responses in these particular population has not been determined yet. Fourthly, clinical outcomes including the infection rate, COVID-19 hospitalization, the occurrence of severe cases, and mortality were vital of future researches in heterologous vaccination. Lastly, waning of immune responses has been observed after COVID-19 vaccination, with reduced protection against infection and some loss of protection against hospitalization and death. Thus, comparison between heterologous and homologous COVID-19 booster vaccination in individuals who have completed full course of vaccination is encouraged.

Our systematic review is not without limitations. First, we didn't make the meta-analyses and pooled evidence for immunogenicity of different studies due to inconsistency of various evaluation indicators and measurement methods in limited included studies. Despite this, we tried to obtain deterministic conclusions by qualitative synthesis analyses. Second, the number of studies and sample size of subjects available in some evaluated strategies was small. The language restriction to English may narrow the breadth of our search. Third, there were no more available data to assess other heterologous combination styles, such as CoronaVac, ZF2001, etc. Fourth, the efficacy of different heterologous schedules for individuals with underlying medical conditions in this meta-analysis was not considered. Thus, the conclusion should be extrapolated carefully in individuals with underlying medical conditions. Further studies are necessary to explore optimal heterologous schedules applicable to those individuals. Fifthly, the comparison on efficacy of different heterologous schedules would provide more valuable information for public health policy decision-making. However, few studies directly compared the efficacy of different heterologous schedules. The inconsistent testing methods of immune response in the included studies limited the indirect comparison. Lastly, all included studies in this review had not reported the clinical efficacies including infection rate, hospitalization rate, and mortality after vaccination. These efficacies should be the crucial evaluation indicators in future.

## Conclusions

Our review showed that heterologous ChAd/BNT schedule induced at least comparable immunogenicity compared with homologous BNT/BNT and better immunogenicity than homologous ChAd/ChAd vaccination. Despite more common adverse in heterologous ChAd/BNT immunisation, they were tolerant, and no serious adverse events were observed. Heterologous ChAd/BNT vaccination is an evidence-based promising vaccination schedule for combating the COVID-19 pandemic.

## Supplementary Information

**Additional file 1: Table S1.** Assessment of quality of the included observational studies (Newcastle–Ottawa Quality Assessment Scale). **Table S2.** Collection methods of adverse events and the frequencies of specific adverse events in the included studies. **Table S3.** Testing methods of anti-SARS-CoV-2 IgG, Neutralizing Antibodies, and cellular immunity in the included studies

## Data Availability

All data generated or analysed during this study are included in this published article and its Additional files.
