# Supplementary material for: Immunogenicity and safety of heterologous versus homologous prime-boost schedules with an adenoviral vectored and mRNA COVID-19 vaccine: a systematic review
Source: Infect Dis Poverty. 2022 May 13;11:53. doi: 10.1186/s40249-022-00977-x (PMC9100319; doi:10.1186/s40249-022-00977-x)
Supplement: Supplementary file 1 — Additional file 1: Table S1. Assessment of quality of the included observational studies (Newcastle–Ottawa Quality Assessment Scale). Table S2. Collection methods of adverse events and the frequencies of specific adverse events in the included studies. Table S3. Testing methods of anti-SARS-CoV-2 IgG, Neutralizing Antibodies, and cellular immunity in the included studies [file 40249_2022_977_MOESM1_ESM.docx]

**Supplementary Material Table S1.** Assessment of quality of the included observational studies (Newcastle-Ottawa Quality Assessment Scale).

| First author (year) | a | b | c | d | e | f | g | h | i | Total |
| --- | --- | --- | --- | --- | --- | --- | --- | --- | --- | --- |
| Louise Benning et al (2021) | 0 | 1 | 1 | 1 | 0 | 0 | 1 | 1 | 1 | 6 |
| Joana Barros-Martins et al (2021) | 0 | 1 | 1 | 1 | 0 | 0 | 1 | 1 | 1 | 6 |
| Alexandre Vallée et al (2021) | 0 | 1 | 1 | 1 | 1 | 1 | 1 | 1 | 1 | 8 |
| Tina Schmidt et al (2021) | 0 | 1 | 1 | 1 | 0 | 0 | 1 | 1 | 1 | 6 |
| David Hillus et al (2021) | 0 | 1 | 1 | 1 | 0 | 0 | 1 | 1 | 1 | 6 |
| Dorit Fabricius et al (2021) | 0 | 1 | 1 | 1 | 0 | 0 | 1 | 1 | 1 | 6 |
| Matthias Tenbusch et al (2021) | 0 | 1 | 1 | 1 | 0 | 0 | 1 | 1 | 1 | 6 |
| Swantje I. Hammerschmidt et al (2021) | 0 | 1 | 1 | 1 | 0 | 0 | 1 | 1 | 1 | 6 |
| Rudiger Groß, et al (2022) | 0 | 1 | 1 | 1 | 1 | 1 | 1 | 1 | 1 | 8 |
| Bruno Pozzetto, et al (2021) | 0 | 1 | 1 | 1 | 1 | 1 | 1 | 1 | 1 | 8 |
| Samantha J Westrop, et al (2022) | 0 | 1 | 1 | 1 | 1 | 1 | 1 | 1 | 1 | 8 |
| Swantje I. Hammerschmidt, et al (2022) | 0 | 1 | 1 | 1 | 0 | 0 | 1 | 1 | 1 | 6 |

1. Representativeness of the exposed cohort;
2. Selection of the non-exposed cohort;
3. Ascertainment of exposure;
4. Demonstration that outcome of interest was not present at start of study;
5. Comparability of cohorts on the basis of the design or analysis (adjusted for age);
6. Comparability of cohorts on the basis of the design or analysis (adjusted for other);
7. Assessment of outcome;
8. Was follow-up long enough for outcomes to occur;
9. Adequacy of follow-up of cohort

**Supplementary Material Table S2.** Collection methods of adverse events and the frequencies of specific adverse events in the included studies

| First Author | Collection methods | Vaccination  Schedule | Local events | | | Systemic events | | | | | | |
| --- | --- | --- | --- | --- | --- | --- | --- | --- | --- | --- | --- | --- |
|  |  |  | Pain | Redness | Swelling | Headache | Myalgia | Fatigue | Arthralgia | Feverishness and chills | Nausea, vomiting | Diarrhoea |
| Louise Benning^[21]^ | Using a 12-item questionnaire inquiring side-effects after vaccination and the use of pain medication after vaccine reception. | ChAd/ChAd | NR | NR | NR | NR | NR | NR | NR | NR | NR | NR |
|  |  | BNT/BNT | NR | NR | NR | NR | NR | NR | NR | NR | NR | NR |
|  |  | ChAd/BNT | NR | NR | NR | NR | NR | NR | NR | NR | NR | NR |
| Xinxue Liu^[14]^ | During the baseline visit, participants were given an oral thermometer, tape measure, and diary card (electronic or paper) to record solicited, unsolicited, and medically attended AEs with instructions. The study sites’ physicians reviewed the diary card regularly to record AEs, AEs of special interest, and SAEs. Abnormal clinical findings from medical history or examination will be assessed. | ChAd/ChAd | NR | NR | NR | NR | NR | NR | NR | NR | NR | NR |
|  |  | BNT/BNT | NR | NR | NR | NR | NR | NR | NR | NR | NR | NR |
|  |  | ChAd/BNT | NR | NR | NR | NR | NR | NR | NR | NR | NR | NR |
|  |  | BNT/ChAd | NR | NR | NR | NR | NR | NR | NR | NR | NR | NR |
| Tina Schmidt ^[26]^ | Local and systemic adverse events within 7 days after vaccination were self-reported using a standardized questionnaire. Reactogenicity after the second dose was collected prospectively in all cases using a standardized questionnaire. Reactogenicity data after the first dose were collected retrospectively in the majority of cases, but all participants felt confident in recalling AEs at the time of enrollment into the study. | ChAd/ChAd | NR | – | NR | NR | NR | NR | NR | NR | NR | NR |
|  |  | BNT/BNT | NR | – | NR | NR | NR | NR | NR | NR | NR | NR |
|  |  | ChAd/BNT | NR | – | NR | NR | NR | NR | NR | NR | NR | NR |
| David Hillus^[13]^ | Participants were asked to fill in electronic questionnaires on reactogenicity,AEs, medication, and medical visits on days 1, 3, 5, and 7 after the first and second vaccination. | ChAd /ChAd | 53(37–68) | 3(0.1–14.2) | 3(0.1–14.2) | 22(11.7–38.1) | 14(6.1–28.7) | 33(20.2–49.7) | 19(9.8–35) | 22(11.7–38.1) | 3.0(0.1–14.2) | 6(1–18.1) |
|  |  | BNT/BNT | 70(62.3–76.4) | 2(0.5–5.4) | 8(4.4–12.7) | 39(31.8–46.7) | 34(27.1–41.6) | 48(40.2–55.5) | 10(6.3–15.7) | 20(14.1–26.3) | 4(2.1–8.8) | 6(3.5–11.2) |
|  |  | ChAd /BNT | 79(70–85.6) | 7(3.3–13.2) | 7(3.3–13.2) | 35(26.2–44.2) | 20(13.6–28.9) | 36(27–45.1) | 7(3.3–13.2) | 18(12–26.8) | 6(2.7–12) | 4(1.5–9.5) |

NR indicates the study did not report definite number, frequency, or statistics test results.

Abbreviations: AE, adverse events;SAE, serious adverse events

**Supplementary Material Table S3.** Testing methods of anti-SARS-CoV-2 IgG, Neutralizing Antibodies, and cellular immunity in the included studies

| First Author | RBD Ab | Spike protein Ab | Neutralizing Ab | T cell response and others |
| --- | --- | --- | --- | --- |
| Louise Benning^[21]^ | A multiplex bead-based assay for the Luminex platform (LabScreen COVID Plus) was performed (One Lambda Inc., West Hill, CA, USA). The assay detects participant’s antibody response to 5 different SARS-CoV-2 proteins, namely, the full spike protein, 3 individual domains of the spike protein (S1, S2, and RBD), and the nucleocapsid. | A multiplex bead-based assay for the Luminex platform (LabScreen COVID Plus) was performed (One Lambda Inc., West Hill, CA, USA). The assay detects participant’s antibody response to 5 different SARS-CoV-2 proteins, namely, the full spike protein, 3 individual domains of the spike protein (S1, S2, and receptor binding domain), and the nucleocapsid. | A plate-based SARS-CoV-2 sVNT (Medac, Wedel, Germany) was used to identify the binding-inhibition potency of serum samples. The test is based on antibody-mediated blockade of the interaction between the ACE2 receptor protein and the RBD of the SARS-CoV-2 spike protein and mimics the virus–host interaction by direct protein–protein interaction. | ­– |
| Joana Barros-Martins^[12]^ | – | To determine SARS-CoV-2 IgG serology by quantitative ELISA (anti-SARS-CoV-2 S1 spike protein domain IgG SARS-CoV-2 QuantiVac, Euroimmun) according to the manufacturer’s instructions (dilution 1:400 or 1:600) | To determine neutralizing antibodies against the Wuhan spike, the B.1.1.7-spike (Alpha), the P.1-spike (B.1.1.28.1; Gamma) and the B.1.351-spike (Beta) variants of SARS-CoV-2-S in plasma. In this assay, the soluble receptor for SARS-CoV-2—ACE2—is bound to 96-well-plates to which different purified tagged RBDs of the spike protein of SARS-CoV-2 can bind once added to the assay. | T cell re-stimulation: cells were diluted with equal volumes of peptide pools containing S-protein or mixture of M-, N- and E-proteins. Flow cytometric analysis of spike-specific B cells. Quantification of IFN-γ and TNF-α release by the LEGENDplex kit (BioLegend) according to the manufacturer’s instructions. |
| Xinxue Liu^[14]^ | – | Serum samples were analysed at Nexelis (Laval, Canada) to determine SARS-CoV-2 anti-spike IgG concentrations by ELISA (reported as ELISA laboratory units [ELU]/mL) | The NT_50_ for SARS-CoV-2 pVNT was tested using a vesicular stomatitis virus backbone adapted to bear the SARS-CoV-2 spike protein | IFNγ-secreting T cells specific to whole spike protein epitopes designed based on the Wuhan-Hu-1 sequence (YP_009724390·1) were detected using a modified T-SPOT-Discovery test. T-cell frequencies were reported as SFC per 250 000 PBMCs with a lower limit of detection of one in 250 000 PBMCs. |
| Alexandre Vallée^[28]^ | – | SARS-CoV-2 IgG II Quant assays were performed on the Abbott Alinity i platform in accordance with the manufacturer’s package insert. | – | – |
| First Author | RBD Ab | Spike protein Ab | Neutralizing Ab | T cell response and others |
| David Hillus^[13]^ | To assessed the presence of SARS-CoV-2-specific RBD antibodies using a microarray-based immunoassay (SeraSpot Anti-SARS-CoV-2 IgG, Seramun Diagnostica, Heidesee, Germany). | To assessed the presence of SARS-CoV-2-specific full spike and S1 subunit antibodies using a microarray-based immunoassay (SeraSpot Anti-SARS-CoV-2 IgG, Seramun Diagnostica, Heidesee, Germany). | To investigated the functional neutralization capacity using an RBD-ACE2 binding inhibition assay (sVNT; cPass, medac, Wedel, Germany); Using a SARSCoV-2 pVNT to test the neutralising capacity of the vaccine regimes by determining serum 50% inhibitory dilutions (ID_50_). | SARS-CoV-2 spike-specific T-cell responses were measured by IFN-γrelease assay (IGRA; Euroimmun Medizinische Labordiagnostika) of S1 peptide-stimulated T-cells in whole blood. |
| Dorit Fabricius^[15]^ | – | The EUROIMMUN anti-SARS-CoV-2 ELISA assays (EUROIMMUN, Lübeck, Germany) were used for the detection of IgG and IgA against the S1 domain of the SARS-COV-2 spike (S) protein. | The principle of the blocking ELISA mimics the virus neutralization process, and qualitatively detects anti-SARS-CoV-2 antibodies, which suppress the interaction between RBD fragments of the viral spike (S) protein and ACE2 protein bound to the surface of a microtiter plate | The SARS-CoV-2 IFN-γrelease assay (IGRA, EUROIMMUN, Lübeck, Germany) was used to detect T-cell-mediated immune response to the SARS-CoV-2 spike antigen in EDTA blood. |
| Matthias Tenbusch^[27]^ | – | – | A sVNT (NAb assay; Yhlo, Shenzen, China) based on the competition of serum antibodies with recombinant ACE2 for binding to the SARS-CoV-2 spike protein RBD. | – |
| Swantje I. Hammerschmidt^[23]^ | – | – | pVNT: rhabdoviral pseudotyped particles were produced in 293T cells transfected to express the spike protein of the Delta variant;  sVNT for SARS-CoV-2 variants: in this ELISA-based test, the soluble receptor for SARS-CoV-2, ACE2, is bound to 96-well-plates to which a RBD of the Delta Spike-protein with a C-terminal His-Tag binds once added to the plates. | – |
| First Author | RBD Ab | Spike protein Ab | Neutralizing Ab | T cell response and others |
| Tina Schmidt^[26]^ | SARS-CoV-2-specific IgG antibodies towards the RBD of SARS-CoV-2 spike protein were quantified using ELISA according to the manufacturer’s instructions (SARS-CoV-2-QuantiVac, Euroimmun). | – | A neutralization assay based on antibody-mediated inhibition of soluble ACE2 binding to the plate-bound S1 RBD was used at a single serum dilution according to the manufacturer’s instructions (SARS-CoV-2-NeutraLISA, Euroimmun). | SARS-CoV-2-specific T cells were determined from heparinized whole blood after 6-h stimulation with overlapping peptides spanning the SARS-CoV-2 spike protein (N-terminal RBD and C-terminal portion including the transmembrane domain, JPT). Immunostaining was performed using anti-CD4, anti-CD8, anti-CD69, anti-IFN-γ, anti-IL-2, and anti-TNF-α. |
| Rudiger Gross^[22]^ | – | IgG and IgA levels in serum were determined by antiSARS-CoV-2 assay (Euroimmun), an ELISA which detects antibodies against the SARS-CoV-2 S1 spike domain. | Prevention of SARS-CoV-2 spike RBD interaction with ACE2 by sera was evaluated by SARS-CoV-2 sVNT Kit (GenScript) according to the manufacturer’s instructions. | Determination of CD4+ and CD8+ SARS-CoV-2 spikespecific T cell responses by ICS |
| Bruno Pozzetto^[25]^ | RBD-specific IgG were measured using bioMérieux Vidas SARS-CoV-2 IgG diagnosis kits | S1-specific IgG were measured using Siemens Atellica IM SARS-CoV-2 IgG (sCOVG); IgA binding to the SARS-CoV-2 spike protein were measured by ELISAs. | A (PRNT was used for the detection and titration of neutralizing antibodies. | Monitoring of T cell responses using whole-blood IFNγ release  assay; monitoring of T cell responses and SARS-CoV-2 RBD-specific B cells response by flow cytometry. |
| Samantha J Westrop^[29]^ | – | IgG antibody levels against the SARS-CoV-2 spike protein (S Ab) was determined using the Roche Elecsys S. | – | – |
| Swantje I. Hammerschmidt^[24]^ | – | – | a sVNT. The sVNT is an ELISA-based assay that quantitatively determines the ability of serum antibodies to inhibit a tagged spike RBD to bind to coated ACE2 that serves as a cellular receptor for SARS-CoV-2. | – |

Abbreviations: Ab, antibody; RBD, receptor-binding domain; ACE2, angiotensin-converting enzyme 2; CMIA, chemiluminescent microparticle immunoassay; IFN-γ, interferon-γ; ELISA, enzyme-linked immunosorbent assay; sVNT, surrogate virus neutralization test; PRNT, plaque reduction neutralization test; ICS, intracellular cytokine staining; SFC, spot forming cells; pVNT, Pseudotyped virus neutralization assay; ID_50_, 50% inhibitory dilutions; NT_50_, 50% neutralising antibody titre
